# Unveiling a Therapeutic Breakthrough: Baricitinib in the Treatment of Acute and Recurrent Pustulosis Associated with Atopic Dermatitis—A Case Report, Literature Review, and Novel Clinical Insights

**DOI:** 10.3390/life15040507

**Published:** 2025-03-21

**Authors:** Daciana Elena Brănișteanu, Cătălina Anca Munteanu, Daniel Constantin Brănișteanu, Cristina Colac-Boțoc, Roxana Paraschiva Ciobanu, Antonia Elena Huțanu, Cătălina Onu-Brănișteanu, Gabriela Stoleriu, Laura Endres, Cojocaru Elena, Stefan Vasile Toader

**Affiliations:** 1 Discipline of Dermatology, Grigore T. Popa University of Medicine and Pharmacy, 16 Universitatii Str., 700115 Iasi, Romania; daciana.branisteanu@umfiasi.ro; 2 Dermatology Clinic, University Clinical Railways Hospital, 1 Garabet Ibraileanu Str., 700115 Iasi, Romania; cristina.botoc28@yahoo.com (C.C.-B.); r.p.ciobanu@gmail.com (R.P.C.); antoniaclivet@yahoo.com (A.E.H.); 3 Discipline of Ophtalmology, Grigore T. Popa University of Medicine and Pharmacy, 16 Universitatii Str., 700115 Iasi, Romania; daniel.branisteanu@umfiasi.ro; 4 Ophtalmology Clinic, University Clinical Railways Hospital, 1 Garabet Ibraileanu Str., 700115 Iasi, Romania; 5 Department of Vascular Surgery, St. Pantelimon Hospital, Pantelimon Str., 021659 Bucharest, Romania; catalina.branisteanu@umfiasi.ro; 6 Faculty of Medicine and Pharmacy, ‘Dunărea de Jos’ University of Galați, 800008 Galați, Romania; stoleriugabriela@yahoo.com; 7 Department of Psycho-Neurosciences and Recovery, Faculty of Medicine and Pharmacy, University of Oradea, 410073 Oradea, Romania; laura_endres@yahoo.com; 8 Department of Morphofunctional Sciences I, Grigore T. Popa University of Medicine and Pharmacy, 700115 Iasi, Romania; elena2.cojocaru@umfiasi.ro; 9 Discipline of Physiopathology, Grigore T. Popa University of Medicine and Pharmacy, 16 Universitatii Str., 700115 Iasi, Romania; stefan.toader@umfiasi.ro

**Keywords:** non-microbial pustules, monomorphic pustules, atopic dermatitis, JAK inhibitors, sun exposure, skin biopsy, inflammatory infiltrate, actinic folliculitis, superficial actinic folliculitis, acne aestivalis

## Abstract

Acute and recurrent pustulosis (ARP), previously known as actinic folliculitis, superficial actinic folliculitis, or even acne aestivalis, is a rare, underdiagnosed dermatological condition characterized by the sudden onset of monomorphic pustular eruptions on an erythematous background localized predominantly on the upper body. While typically associated with sun exposure, ARP can also be triggered by other factors, such as heat or sweating, underscoring its multifactorial etiology. We report the case of a 40-year-old woman with ARP, presenting diagnostic challenges due to overlapping clinical features and the coexistence of atopic dermatitis (AD), an association not previously documented in the literature. The patient exhibited recurrent pustular episodes localized on sun-exposed and non-exposed areas, unresponsive to conventional therapies. Comprehensive microbiological, histopathological, and clinical assessments excluded infectious, drug-induced, and other inflammatory pustular dermatoses, confirming the diagnosis of ARP. Importantly, treatment with Baricitinib, a Janus kinase (JAK) inhibitor primarily prescribed for AD, resulted in marked improvement in both conditions, suggesting shared inflammatory pathways. This therapeutic response highlights the potential role of JAK inhibitors in ARP management, particularly in cases resistant to standard interventions. This report also proposes the inclusion of heat- and sweat-induced ARP as a distinct subtype, expanding the understanding of its diverse triggers beyond UV radiation. Furthermore, it underscores the need for standardized diagnostic criteria and a structured approach to differential diagnosis, given the condition’s underdiagnosed and often misinterpreted nature. By shedding light on the clinical and therapeutic aspects of ARP, this case contributes to a more nuanced understanding of this rare entity and its potential interplay with inflammatory skin disorders such as AD.

## 1. Introduction

Acute and recurrent pustulosis (ARP) is a rare, controversial entity, previously described in the literature as both actinic folliculitis (AF) and superficial actinic folliculitis (ASF). ARP manifests as a non-microbial, episodic pustular eruption, characterized by multiple pustules arranged on an erythematous background [1,2]. The association between ARP and atopic dermatitis is unusual and has not been previously reported in the literature. The positive response of both conditions to Baricitinib, initially intended for AD treatment, highlights the inflammatory nature of ARP, supporting the hypothesis of a potential comorbidity with AD.

## 2. Case Report

**Figure 1:**
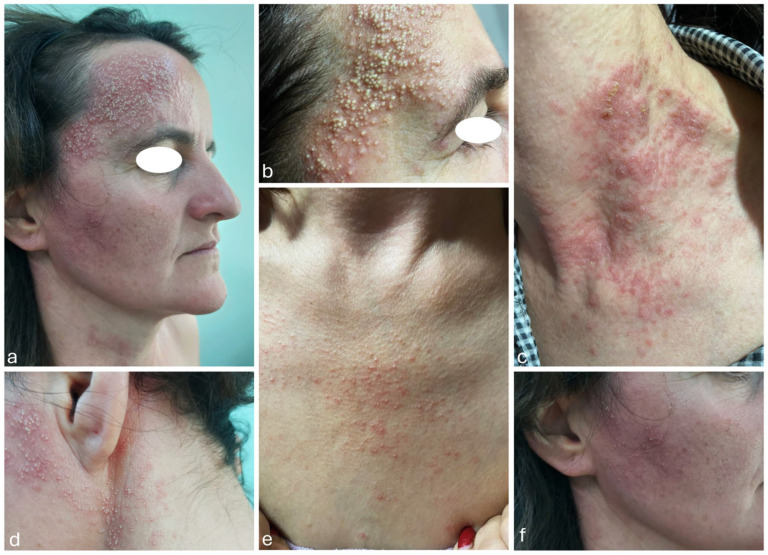
Multiple small monomorphic pustules localized on the face (**a**,**b**,**f**), as well as on the anterior thoracic region, retroauricular area, and anterior cervical region (**c**,**d**,**e**).

We present the case of a 40-year-old Caucasian woman (Figure 1) that was admitted to our dermatology department in 2022, presenting with multiple monomorphic pustules of small size, located on an erythematous background, mildly pruritic, mainly localized on the bilateral temporal region (Figure 1a), forehead (Figure 1b), anterior thoracic region (Figure 1c), bilateral retroauricular (Figure 1d), anterior cervical region (Figure 1e) and bilateral zygomatic region (Figure 1f).

A thorough medical history revealed that the initial lesions appeared approximately two years prior, with recurrences occurring three to four times per year. Although flare-ups correlated with a confirmed history of sun exposure, some episodes occurred independently of it. The hereditary collateral history includes two daughters with atopic dermatitis, one of whom is currently undergoing systemic biological treatment with Dupilumab. The only personal comorbidities worth mentioning are allergic rhinitis, atopic-type skin manifestations, chronic hand dermatitis, and generalized hyperhidrosis.

Initially, due to its suggestive appearance, we raised the suspicion of acute localized exanthematous pustulosis (ALEP), which is actually a variant of acute generalized exanthematous pustulosis (AGEP). However, we were unable to identify any medication or antibiotic to single out, beyond the positive history of recent sun exposure. It is important to mention that our patient is a housewife who spends a lot of time gardening in the sun without any form of protection. She reported that until the age of 38, she had never experienced this type of eruption, despite frequent unprotected sun exposure.

Given the uncertainty of the diagnosis, we performed extensive blood tests, multiple bacteriological cultures from the pustules, cutaneous parasitological examination for Demodex, and mycological examinations. The blood tests revealed no significant abnormalities, and the microbiological, mycological, and parasitological examinations were negative. The patient was discharged with a strong recommendation to use topical sunscreen with SPF 50+, oral photoprotection with capsules based on *Polypodium leucotomos*, and to wear long-sleeved clothing and a wide-brimmed hat during sun exposure.

After a year of absence, the patient returned during a significant flare-up, reporting a recurrence approximately three times during this period, this time without a positive history of sudden sun exposure. The patient stated that she had strictly followed all our recommendations. Even so, the patient reported experiencing several episodes of excessive sweating and exposure to excessive heat during household chores, circumstances that, each time, led to the onset of a new flare-up. The lesions were more severe and, most importantly, appeared in previously unaffected areas, including the abdomen, scalp, and posterior cervical region.

During the same hospitalization, a diagnosis of atopic dermatitis was established, a diagnosis that had been suspected since the first admission. Based on the appearance and location of some of the lesions (Figure 1b), the initial differential diagnoses considered were allergic contact dermatitis and secondary infection of atopic dermatitis lesions. However, these were later excluded by correlating the clinical presentation (the presence of pustules instead of vesicles) with the fact that the pustules were non-microbial.

**Figure 2:**
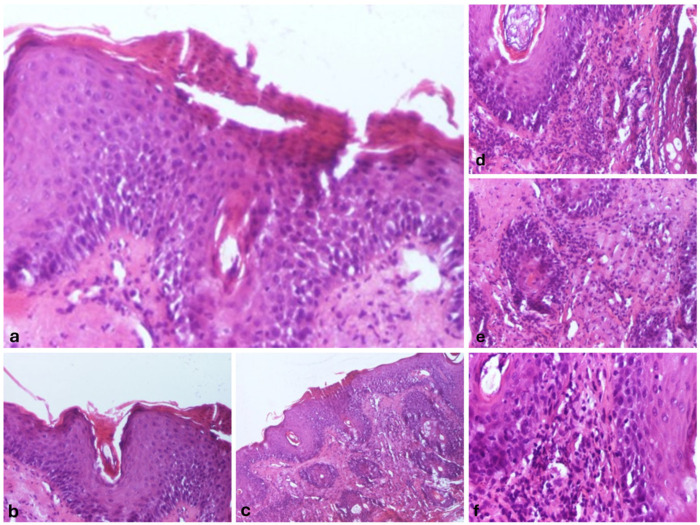
Morphopathological features revealed the following: epidermis with orto- and parakeratosis, Hematoxylin and Eosin (HE), 100× magnification (**a**); follicular hyperkeratosis, with keratinous material filling the infundibulum of the hair follicles, HE, 100× magnification (**b**); epidermis with irregular acanthosis, and focal spongiosis, HE, 40× magnification (**c**); superficial perivascular and perifollicular infiltrate, primarily composed of lymphocytes, with occasional neutrophils, HE, 100× magnification (**d**); follicular epithelium damage with the dermal region surrounding the follicle infiltrated by a mixture of lymphocytes and neutrophils, HE, 100× magnification (**e**); and perivascular and perifollicular infiltrate, primarily composed of lymphocytes, with occasional neutrophils, HE, 100× magnification (**f**).

We also performed a skin biopsy (Figure 2) that revealed an ortokeratotic epidermis with focal areas of parakeratosis at the follicular openings (Figure 2a,b), irregular acanthosis, and focal spongiosis (Figure 2c). Numerous pilosebaceous follicles were observed, surrounded by a dense inflammatory infiltrate composed of lymphocytes and neutrophils, along with their infiltration into the surrounding tissue (Figure 2d–f).

Given the moderate-to-severe form of atopic dermatitis (SCORAD = 45, DLQI = 24) and the lack of efficacy of topical therapy, oral corticosteroids were initially prescribed. After two months, SCORAD remained at 40, and the patient’s quality of life was severely affected by both atopic dermatitis and ARP lesions. According to the national protocol in our country, we decided to initiate advanced systemic therapies, with Dupilumab and JAK inhibitors being the only reimbursed options. After careful consideration, including literature review and patient preference for oral therapy, we chose to start Baricitinib 4 mg/day. The patient had no contraindications or comorbidities that would preclude the use of a JAK inhibitor, making it a feasible option. Remarkably, the results on the eczema lesions were spectacular, with significant improvement being observed.

**Figure 3:**
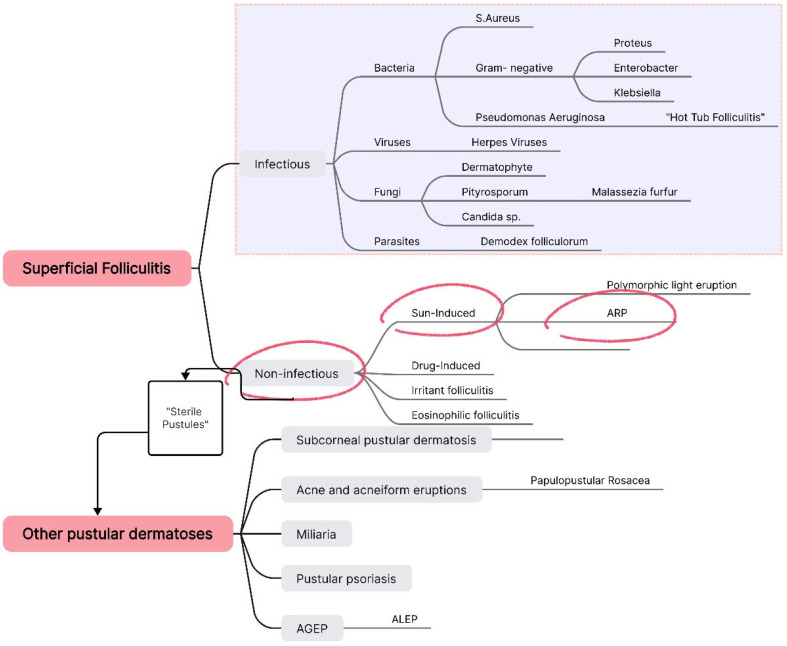
Diagnostic algorithm for ARP, based on the systematic exclusion of all superficial folliculitis types and other pustular dermatoses.

At this point, we focused exclusively on diagnosing the pustular lesions, systematically reviewing each type of superficial folliculitis and other possible pustular pathologies. We employed a straightforward method based on exclusion, until reaching a final diagnosis of acute and recurrent pustulosis (Figure 3).

We initially ruled out superficial infectious folliculitis by conducting bacteriological cultures, Tzanck cytodiagnosis, parasitological skin examination, and mycological evaluation. Subsequently, we excluded any drug-induced folliculitis along with AGEP and ALEP due to the absence of systemic manifestations, with the patient also reporting not having taken any medications or dietary supplements in recent years. Pathologies such as eosinophilic folliculitis, subcorneal pustular dermatosis, and atypical forms of pustular psoriasis were also eliminated based on the absence of specific clinical and paraclinical findings, as well as the lack of characteristic histopathological features.

We focused on sun-induced dermatoses, particularly due to the patient’s history of recent sun exposure and recurrence of pustular episodes triggered by sun contact. We ruled out the possibility of polymorphic light eruption (PMLE), considering that the patient’s lesions were both follicular and monomorphic—characteristics that contrast with PMLE. We arrived at a final diagnosis of acute, recurrent pustulosis, triggered, in this case, by both sun exposure and excessive heat/sweating, in a patient with an underlying atopic predisposition.

**Figure 4:**
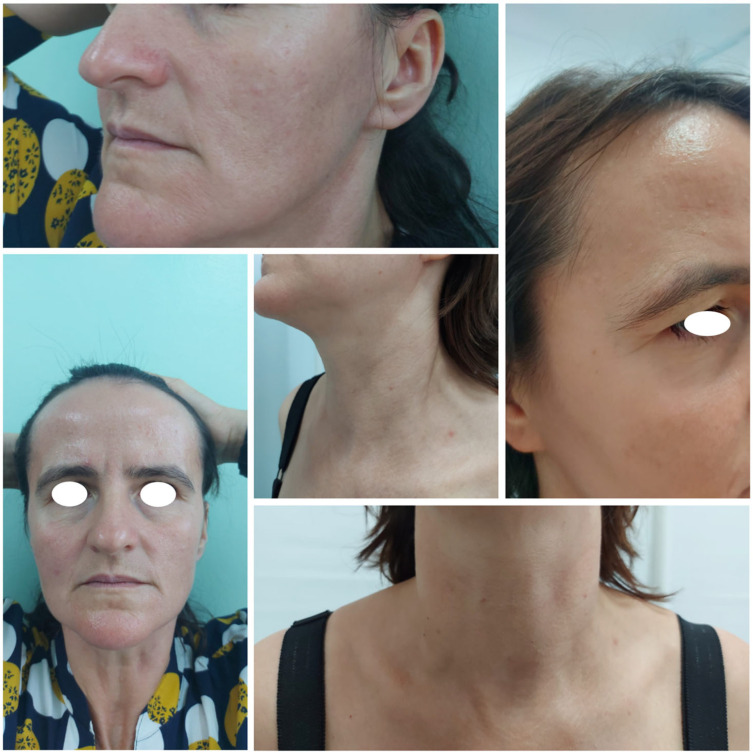
Patient one year after initiating Baricitinib, showing sustained remission of ARP lesions.

Although we applied the same chemical and physical sun protection methods, this time we observed a remarkable reduction in flare-ups and, most importantly, their severity (Figure 4). The only change in the patient’s therapeutic plan was the introduction of Baricitinib for atopic dermatitis, with no modifications to the ARP treatment.

## 3. Discussion and Review of Literature

The involvement of atopic dermatitis (AD) plays a crucial role in the pathogenesis of ARP in our case. To our knowledge, this is the first case reported in the literature describing the coexistence of atopic dermatitis and ARP in the same patient. Furthermore, it is also the only reported case where Baricitinib, used to treat a concomitant condition, has been proven to reduce the frequency of ARP flares until their complete remission.

AD skin is characterized by the overexpression of Th2 and Th22 cytokines, which contribute to skin barrier dysfunction by altering protein and lipid content [3,4]. The impaired lipid barrier function, marked by reduced levels of ceramides, cholesterol, and free fatty acids in the stratum corneum, compromises the skin’s ability to retain moisture and protect against environmental aggressors such as UV radiation [4,5]. This lipid deficiency not only exacerbates trans-epidermal water loss but also increases susceptibility to allergens, irritants, and microbial colonization, further intensifying the inflammatory response [3,4,5].

Specifically, interleukin-4 (IL-4) and IL-13 have been shown to significantly reduce the expression of proteins essential for the formation of the cornified envelope (such as filaggrin, loricrin, and involucrin) [6,7]. Inhibiting IL-4 results in the re-establishment of these key regulators’ expression levels, a fact that underscores the role of type 2 cytokines in modulating epidermal barrier integrity in atopic dermatitis [5,6,7].

That being said, by inhibiting JAK1 and JAK2, Baricitinib effectively diminishes the signaling of IL-4 and IL-13, leading to a reduction in the inflammatory response. [8] In addition to its effects on these cytokines, Baricitinib also impacts other pro-inflammatory cytokines, resulting in an overall decrease in skin inflammation [9].

There is a high probability in our case that ARP is a consequence of atopic skin, due to alterations in its quality. At this time, we cannot definitively state the relationship between the two conditions, but we can support the inflammatory nature of ARP through its response to the administration of a JAK inhibitor.

Even though ARP has acquired multiple names over time, its clinical, paraclinical, and histopathological manifestations have not significantly changed.

Since Verbov first introduced the term “actinic folliculitis” in 1985, the classification of this sun-induced condition has been a subject of debate [10]. He described it as incorporating features of both acne aestivalis (AA), initially reported by Hjorth et al. in 1979, and ASF, first identified by Niebor in 1982 [11,12]. The continued use of these overlapping terms has contributed to diagnostic uncertainty. In 1989, Norris and Hawk further documented two cases of a papular–pustular facial eruption occurring within 6 to 24 h post-sun exposure and adopted the term ‘actinic folliculitis’ as a unifying diagnosis [13].

Only two authors in the literature mention the term “acute and recurrent pustulosis” (ARP) to actually refer to three other entities grouped under the same umbrella: AA, actinic folliculitis (AF), and ASF [1,2].

In 2017, Guzman et al. proposed this new pathology, presenting three cases and observing multiple common characteristics. All patients were female, and the eruptive episodes appeared suddenly as multiple small pustules on an erythematous background, distributed on the forehead, cheeks, chin, and neck. The recurrence of the condition was demonstrated by its reappearance 4–6 times a year. Although ARP was most commonly overlapped with the term actinic folliculitis in the past, in this case, for the first time, the author emphasizes that a history of sun exposure is not necessary to establish the diagnosis. For this reason, the author proposed the description of three subtypes of ARP, based on the causative agent: (A) UV radiation-induced ARP, (B) infection-induced ARP, and (C) idiopathic ARP [1].

Similarly, in 2023, Porter et al. conducted a review of literature focusing on identifying the uncommon causes of follicular pustulosis induced by UV radiation as well as other factors. They included in their search nine articles describing 23 patients who shared an acute onset and follicular eruptions that spontaneously healed within a maximum of 3 weeks without leaving scars, and recurred over time. Articles with cases that required treatment, where the pustules were of non-neutrophilic origin, were associated with infections, or lasted over 3 weeks were excluded.

They suggested establishing the following specific diagnostic criteria to enhance the likelihood of a quicker and more precise diagnosis: (A) Clinically, it is defined as an acute eruption of monomorphic pustules distributed on an erythematous background, predominantly affecting the head, neck, or anterior chest. (B) Histopathologically, the presence of a perifollicular neutrophilic infiltrate is necessary. (C) The resolution duration of each episode should not exceed 7 days, without treatment. (D) Healing should occur with restitutio ad integrum [2].

Nieboer et al. were the first to attempt to describe the pathogenesis of ARP, hypothesizing that local heat might play a role and suggesting an aberrant ending of eccrine sweat ducts into the follicle [12]. However, this hypothesis was refuted by Jaeger et al. in 2003 through serial histopathological examinations of a patient on the fifth day of the eruption and 48 h after controlled exposure to a temperature of 90 degrees (sauna) followed by UV exposure [14].

Most authors have concluded that the pathogenesis of ARP likely involves a combination of UVA radiation, local heat, and hyperhidrosis. UV radiation is known to induce epidermal thickening, which can lead to follicular infundibular thickening. This glandular occlusion results in inflammation and lesions, resembling other papulopustular eruptions [10,11,12,13,14,15,16]. Unfortunately, this mechanism of action can only account for the UV-induced form of ARP.

Considering that there are documented cases in the literature occurring independently of sun exposure or excessive heat—referred to as idiopathic cases—the precise mechanism underlying ARP remains unknown [1,2]. Further in-depth studies are needed to clarify this mechanism.

Even though it is found under multiple names, ARP represents a unique entity, and it would be most accurate not to classify it under the term photodermatosis, as the UV radiation-induced form is only one subtype of this condition.

To our knowledge, this is also the first case induced successively by multiple causative factors: UV radiation, excessive heat, and sweating. However, not every eruption could be associated with a well-defined cause, as some occurred during the peak of the winter season. The only plausible explanation would be the association of ARP with sensitized, atopic skin.

We believe that establishing a diagnostic algorithm, primarily grounded in detailed differential diagnosis, would be valuable. This approach could complement and further support the clinical and paraclinical criteria suggested by Porter et al., facilitating a more accurate final diagnosis.

**Figure 5:**
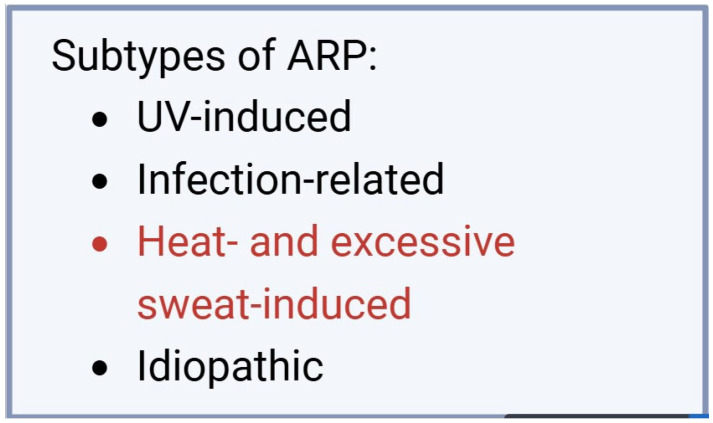
The four ARP subtypes, including the newly proposed subtype, aimed at facilitating diagnosis.

In addition to Guzman et al.’s classification, we propose the inclusion of a new ARP subtype, as shown in Figure 5, which is precipitated by excessive heat and sweating. This is particularly relevant given that other documented cases in the literature have been attributed to this factor.

## 4. Conclusions

This article highlights a rare association not previously reported in the literature and raises awareness of the potential link between ARP and other inflammatory diseases, such as AD in our case.

Additionally, reporting a new case of ARP and proposing a new subtype will aid in facilitating the diagnosis of this condition.

Even though it may be considered a self-limiting disease, multiple recurrences of it can make it severe. Therefore, the focus should be on reducing recurrences and improving patients’ quality of life.

In our case, the patient was much more affected by the pustular lesions than by the atopic dermatitis, as the pustular lesions significantly impacted her self-esteem, being more visible.

It is essential to establish this condition firmly as acute, recurrent pustulosis to avoid interpretative ambiguity regarding its characteristics and to reduce misidentification caused by inconsistent nomenclature. Currently, we consider this pathology underdiagnosed or even misdiagnosed.

Although recent years have seen advancements in diagnosing and elucidating its clinical and paraclinical pathogenetic characteristics, the exact etiology remains unresolved, underscoring the need for further advanced studies in the future.

## Data Availability

All data are reported in the manuscript.

## Abbreviations

The following abbreviations are used in this manuscript:

ARP	Acute and recurrent pustulosis
AD	Atopic dermatitis
UV	Ultraviolet
JAK	Janus kinase
ALEP	Acute localized exanthematous pustulosis
AGEP	Acute generalized exanthematous pustulosis
IL	Interleukin
Th	T-helper cells
PMLE	Polymorphous Light Eruption
ASF	Actinic Superficial Folliculitis
AF	Actinic folliculitis
AA	Acne aestivalis
